# Chronic enteropathy in dogs affects the quality of life in both dogs and their owners—are veterinarians proficient in handling the caregiver burden?

**DOI:** 10.3389/fvets.2024.1488917

**Published:** 2025-01-07

**Authors:** Janne Graarup-Hansen Lyngby, Lise Nikolic Nielsen, Stine Ankerkilde, Amanda Wissendorf Bentzen, Charlotte Bjørnvad, Thomas Bøker Lund, Peter Sandøe

**Affiliations:** ^1^Department of Veterinary Clinical Sciences, University of Copenhagen, Frederiksberg, Denmark; ^2^Department of Veterinary and Animal Sciences, University of Copenhagen, Frederiksberg, Denmark; ^3^Department of Food and Resource Economics, University of Copenhagen, Frederiksberg, Denmark

**Keywords:** canine, animal-owner bond, quality of life, inflammatory bowel disease, chronic gastrointestinal disease, canine chronic enteropathy clinical activity index (CCECAI)

## Abstract

**Introduction:**

Chronic disease is generally known to affect dogs’ quality of life (QoL) as well as being associated with increased strain on their owners. Gastrointestinal (GI) disease is a common problem in companion animal practice, yet little is known about the QoL of dogs with chronic enteropathy (CE) and how their owners and veterinarians assess it.

**Methods:**

The aim of this study was to explore: (i) how dog owners and veterinarians observed and evaluated QoL for dogs with chronic GI disease, (ii) how having a dog with CE affected the owner’s QoL, and (iii) characteristics of the communication and relationship between the dog owner and veterinarian. Twenty owners of dogs with CE and 20 companion animal veterinarians were included in this qualitative, interview-based, exploratory study.

**Results:**

Owners evaluated QoL based on their dog’s apparent emotional state, the presence of clinical signs, or restrictions in their daily life. In their assessments, veterinarians looked at the presence or absence of normal behavior, but also at disease severity and the emotional state of the dog. The majority of owners experienced many concerns and burdens that impacted their own QoL, including daily logistical challenges, implementing therapeutic regimens such as diet restriction, administering multiple daily medications, and the strain of nursing responsibilities on the owner-dog relationship. Dog owners generally felt that communication with their veterinarians was good, while veterinarians found the communication laborious and time-consuming.

**Discussion:**

In general, owners and veterinarians were aligned in their QoL assessments, and the majority of veterinarians relied heavily on the owners’ input and observations. However, assessments were not done in a standardized fashion among either group. Logistical challenges of having a dog with a chronic GI disease often lead to lifestyle changes for the owners, including altering working hours and cancelling holidays or other social arrangements. Having a dog with CE therefore affected the owners’ QoL even when the dogs were clinically stable. Providing owners with written material about the condition in addition to medical and feeding regimen instructions may help the owner, improve compliance, and decrease the non-billable hours the veterinarian must spend communicating with the owner about their dog’s CE.

## 1 Introduction

Gastrointestinal (GI) clinical signs like vomiting and diarrhea are some of the most common complaints in dogs presenting in small animal practice (1–4). While these clinical signs may be self-limiting and responsive to non-specific supportive care, they may also progress into a more chronic state. Chronic enteropathy (CE) is defined as clinical signs with a duration of more than 3 weeks and clinical characteristics that could be related to a range of conditions (5). Hence, the diagnostic process includes a range of laboratory investigations and clinical steps where the owner is heavily involved. These include strictly adhering to dietary elimination trials at home for several weeks, and repeat visits to the veterinarian for blood analyses, diagnostic imaging, and ultimately GI biopsies for histopathology. This is time-consuming and costly for the owner. The ideal treatment varies among dogs and an appropriate treatment regimen is determined by trial and error. The owner is required to follow the veterinarian’s instructions diligently to ensure response to therapy and to avoid a relapse or worsening of clinical signs.

In human medicine, living with a chronic GI disease has been shown to be related to a reduced quality of life (QoL) (6–9), with some studies reporting an increased prevalence of anxiety and depression in this patient group (6, 7, 10). Tools to assess QoL have been applied and evaluated in companion animal populations for a range of diseases (11–17). One recent study assessed the QoL of dogs with CE (18) and found that they generally had a lower overall QoL as well as poorer interactions with their owners compared to dogs with a cancer diagnosis (18).

Having a dog with a chronic disease may also be a strain on the owner. Studies have previously investigated the caregiver’s burden and revealed reduced QoL and decreased psychosocial functioning among owners of dogs with conditions such as chronic dermatological disease (19, 20), cancer (21), and general illness (14, 22). The burden of the primary caregivers of dogs with GI disease specifically has not been investigated to date. However, there is reason to suspect that having a dog with CE is also associated with a decreased QoL for owners due to concern about the wellbeing of their dog as well as logistical challenges related to visits to the veterinarian, medications, treatments, and monitoring. The owners may observe clinical signs or behavior at home that are not recognized by the veterinarian, while the severity and impact on QoL may also be viewed differently by owners and veterinarians. It is therefore important to advance our knowledge about possible discrepancies in how veterinarians and dog owners view QoL for dogs with CE and to highlight possible challenges when veterinarians and owners communicate.

In veterinary medicine, owners may experience increased anxiety over their pets’ chronic illness and require more non-billable contact with the veterinarian (14). Such owner behavior can result in an increased unpaid workload for veterinarians.

These potential human strains and stressors highlight the need for clear, timely, and well-planned communication between veterinarians and caregivers of dogs with chronic conditions so as to alleviate the caregiver’s burden and reduce the need for additional contact with the veterinarian. Communication from the veterinarian therefore needs to be tailored to each owner’s needs and abilities. Specific types of questions should therefore be asked by the veterinarian to reveal how the owner is affected (23, 24), while potential barriers to good communication should be identified. These include time constraints, multiple owners, and language barriers, which have previously been shown to affect veterinarian-client interactions (23).

In this qualitative, interview-based study we therefore set out to explore: (i) how dog owners and veterinarians observe and evaluate QoL for dogs with chronic GI disease, (ii) the impact of having a dog with CE on the owner’s QoL, (iii) and characteristics of the communication and relationship between the dog owner and veterinarian.

## 2 Materials and methods

### 2.1 Participants

Two groups were recruited for the study: 20 dog owners and 20 veterinarians. The owners and veterinarians were eligible for participation if they could speak and understand Danish. An effort was made to recruit both owners and veterinarians in different geographic locations (countryside and urban areas) and from different regions in Denmark.

Dog owners were recruited via a combination of flyers at the University Hospital for Companion Animals, University of Copenhagen (UCPH), at local companion animal practices, and through social media. A number of owners were recruited directly through their own veterinarian. Initially, dogs diagnosed with chronic GI disease in the past 3 months were included, but to facilitate faster recruitment, the inclusion criteria were extended to a diagnosis within the last 5 years. Further inclusion criteria were confirmation of the diagnosis based on histopathology on endoscopically or surgically obtained biopsies within the last 5 years and disease response to dietary and/or medical therapy. Exclusion criteria were any known co-morbidities that could affect the dog’s QoL, and whether the owner suffered from a disease that could affect their perception of the dog’s disease.

Veterinarians were recruited by email correspondence or telephone contact directly by the authors. The veterinarians were included if they worked primarily in companion animal practice and had at least 3 years of experience. We strived to obtain the following distribution in work experience: ten general practitioners from companion animal clinical practice, five general practitioners with a postgraduate, two-year continuing education qualification (equivalent to a general practitioner certificate), and five veterinarians with specialty training (PhD, Diplomate, or Master of Companion Animal Clinical Sciences) within companion animal internal medicine and gastroenterology.

### 2.2 The interview guides

An exploratory qualitative study design was used. Two separate interview guides—one for veterinarians and one for owners—were developed based on reviews of relevant literature and discussions among the two master students, SA and AWB, and their supervisors, CRB, LNN, and PS. Two of the supervisors, CRB and LNN, have many years of experience treating GI patients and discussing the treatment with colleagues and therefore ensured that the perspective from practice was reflected in the interview guide. The interviews were pilot-tested on two owners and one veterinarian with the aim of improving the interviewees’ understanding of the questions. This did not lead to any revisions of the final interview guides, but these participants were nevertheless not included in the final study material.

The interview guide for the owners covered four areas: (i) the course of the GI disease, with a table of clinical signs commonly exhibited by dogs with CE, (ii) the dog’s QoL as perceived by the owner, (iii) the owner’s own QoL when living with a dog with GI disease, and (iv) the owner’s experience of seeking information relating to their dog’s disease and communicating with the veterinarian. In addition, descriptive information regarding the informant’s socio-demographic characteristics was collected.

The interview guide for the veterinarians included two specific areas: (i) their perception of a good QoL for dogs in general and specifically for dogs with GI disease, and (ii) their experience of communicating with the owners of dogs with chronic GI disease. Furthermore, information regarding the veterinarian’s level of experience (new graduate to highly experienced) and the nature of the practice (general vs. referral practice) was collected.

English translations of the interview guides (one for veterinarians and one for dog owners) are provided as Supplementary material (Supplementary file S1).

### 2.3 Data collection and analysis

The interviews were mostly performed face-to-face, either in the owners’ homes or in the veterinarians’ clinical practices (or in one case at a home office). Three owners had interviews conducted online. At the face-to-face interviews, participants received a box of chocolates for their participation. Answers to follow-up questions were obtained through e-mail correspondence in a few instances. The owners and veterinarians were not paired.

Interviews were carried out from September to November 2021 by two of the co-authors (SA and AWB) as part of their veterinary Master’s project. In total, 40 interviews (20 dog owners and 20 veterinarians) were performed.

Interviews were audio recorded using a mobile phone and were transcribed verbatim by SA and AWB. The transcriptions were checked for accuracy against the audio recording, and irrelevant data and repetitions were removed. The transcriptions were also anonymized to the extent possible by removing information that directly or indirectly revealed the identity of the interviewees.

The data analysis design was coordinated and decided by the core author team (JGHL, LNN, CB, TBL, and PS). Qualitative, thematic analysis was performed using abductive data analysis (25). First, JGHL and LNN read through all transcribed interviews with veterinarians and dog owners and became familiar with emerging patterns in order to identify trends across the dataset. Based on the transcripts, JGHL and LNN, who both possess extensive clinical experience regarding treatment of GI disease then jointly proposed a number of overarching themes of interest. These themes included both themes specifically addressed in the interview guide as well as additional themes brought up by the dog owners or the veterinarians interviewed. Together with the core author team it was decided how to combine the themes into relevant codes. On that basis, JGHL and LNN constructed a codebook for each group (veterinarians and owners) that aligned with these themes (see Supplementary file S2). All transcripts were entered into nVivo for data coding (nVivo, v12, QSR International Pty, Ltd., Denver, United States). JGHL and LNN independently coded the first three transcripts from each group, after which they evaluated whether they used a similar coding strategy and whether the two codebooks were comprehensive enough. The codebooks were found to be sufficient, and after resolving some minor coding differences, JGHL and LNN coded all interviews independently. After this, JGHL and LNN went through all coded interviews together and reached agreement on the coding of all major themes. They then drafted the results section based on the codes. Using an iterative process, the core team critically reviewed the major findings (cf. the sub-themes) and the citations backing these up, after which JGHL and LNN made some adjustments. Finally, all authors agreed upon the major findings presented in the results section.

## 3 Results

### 3.1 Demographics

#### 3.1.1 Dog owners and dogs

A summary of demographic information for the dog owners and their dogs is shown in Table 1. The majority of participants in the dog owner group identified as women (19/20, 95%). The dog owners were distributed across four age groups: 18–30 years (4/20, 20%), 31–45 years (5/20, 25%), 46–60 years (6/20, 30%) and > 60 years (5/20, 25%).

**Table 1:** Demographic information about owners and dog populations.

Demographic data (dog owners and their dogs)	
Geographical region:	
East Denmark	10
West Denmark	10
Gender: (Male/Female)	1/19
Income group:	
≤200,000 DKK	1
200,000–300,000 DKK	3
>300,000–500,000 DKK	6
>500,000 DKK	8
Not disclosed	2
Age group (owners):	
18–30 yrs	4
31–45 yrs	5
46–60 yrs	6
>60 yrs	5
Age group (dogs):	
1–3 yrs	5
4–6 yrs	10
7–9 yrs	4
≥10 yrs	1
Sex (dog): male/female	12/8
Time the dogs spent alone per day:	
<3 h	12 (60%)
3–5 h	2 (10%)
6–8 h	5 (25%)
>8 h	1 (5%)

Yrs, years.

The majority of dogs (12/20, 60%) spent <3 h home alone per day during the week. Only one dog spent more than 8 h home alone per day (1/20, 5%). At the time of the interview, the dogs had a median age of 4.75 years (range: 1–12 years), and the majority (15/20, 75%) were < 6 years of age. Disease duration ranged from a few months to several years. According to the owners, most of the dogs with CE were stable in their disease with only 15% of dogs having significant clinical signs at the time of the interviews. The most prevalent clinical signs exhibited during the course of the disease (multiple answers accepted) were vomiting (*n* = 19), behavioral changes (*n* = 14), inappetence (*n* = 14), pain (*n* = 13), diarrhea (*n* = 11), restlessness (*n* = 11), weight loss (*n* = 9), and tenesmus (*n* = 8). According to the owners, the clinical signs with the greatest impact on their dogs’ QoL were vomiting (*n* = 5), a combination of vomiting, pain, diarrhea and inappetence (*n* = 3), or pain (*n* = 3).

#### 3.1.2 Veterinarians

A summary of the demographic information for participating veterinarians can be found in Table 2. The majority of veterinarians identified as women (17/20; 85%), and 55% (11/20) were between 46 and 60 years of age. The study represents a broad distribution of practicing veterinarians across Denmark. Only three veterinarians (15%) had less than 8 years of experience in companion animal clinical practice. Eleven veterinarians (11/20; 55%) were general small animal practitioners without formalized postgraduate clinical training, whereas 4/20 (20%) had a Danish Veterinary Association (DVA) Certificate in Small Animal Diseases, and the remaining five veterinarians (25%) were considered specialists. The specialists all had extensive experience of diagnostic work-up and treating dogs with CE in a referral practice setting.

**Table 2:** Demographic data on veterinarians participating in the interviews.

Demographic data, veterinarians	
Gender: Male/Female	3/17
Age group (veterinarians):	
18–30 yrs	1
31–45 yrs	7
46–60 yrs	11
>60 yrs	1
Geographical region:	
West Denmark	10
East Denmark	10
Postgraduate clinical training:	
Small animal general practice	11
DVA certificate in small animal diseases	4
Specialists/special training	5
Years in small animal practice:	
3–5 yrs	2
6–8 yrs	1
9–15 yrs	6
>15 yrs	11

DVA, Danish Veterinary Association; Yrs, years.

### 3.2 Theme 1: the understanding and assessment of quality of life for dogs with chronic enteropathy

#### 3.2.1 Owner perspective

When asked about their dogs’ QoL, the owners said that they evaluated it on the basis of (i) the dog’s apparent emotional state or (ii) presence of clinical signs or restrictions in their daily lives.

##### 3.2.1.1 Emotional states

Most owners described how they perceived “normal,” positive dog behavior such as a good appetite, playfulness, and active social interactions with other owners and dogs as a reference for good QoL: *“She is happy, she is training, so in that sense her QoL is really good” (Dog owner (DO) 11).* Owners often used human-related adjectives to describe how the dogs felt, such as “being naughty” and “having a glint in their eyes” or “being happy or playful” when they were well: *“… he can easily play a game of tag”* (DO14), while they referred to signs of “shame” or sadness when they were not: *“… he has really withdrawn himself from us, he’s minding his own business, he’s been tired and has only wanted peace and quiet.”* (DO8). Two owners reported that their dogs had changed their general behavior and appeared more depressed than before: *“Can I make her play… because I could not at the beginning. She did not want to play; she did not want to do anything.”* (DO11).

##### 3.2.1.2 Presence of clinical signs or restrictions in daily life

A number of owners described the presence of clinical signs associated with CE such as pain, nausea, discomfort, and a reluctance to exercise as indications of a poor QoL. Examples of slightly reduced QoL in dogs according to their owners included if the dog still had clinical signs of illness, a restriction in feeding their dogs anything they wanted, and constant monitoring and supervision by the owners: *“… she is constantly under supervision, that way, you do not have any freedom.” (DO12)*.

#### 3.2.2 Veterinarian perspective

The interviews with the veterinarians revealed that there was no uniform approach to QoL assessment among the participating veterinarians. When asked specifically about QoL for dogs with CE, the veterinarians’ overall assessment strategies were focused around three themes: (i) the presence or absence of normal behavior, (ii) disease severity, and (iii) the emotional state of the dog.

##### 3.2.2.1 Presence or absence of normal behavior

The veterinarians said that when they assessed QoL they focused on the dog’s ability to perform normal activities such as interacting with their owner, eating normal dog food, drinking water, going for a walk, or playing: *“It is important to me that the dog has the same behavior that it has always had”* (Veterinarian 1 (VET1)). This assessment was made based on a history from the owner as well as an assessment of the dog during the consultation. However, it was not directly evident from the interviews when their assessment was based on the owner’s versus their own observations.

##### 3.2.2.2 Disease severity

The majority of the veterinary participants used the presence and frequency of objective or semi-objective clinical signs of GI disease as parameters for assessing QoL. This could include the presence of pain, nausea, vomiting, diarrhea, tenesmus, weight loss, ascites, and/or fecal incontinence, e.g., “*…frequency and severity of vomiting and diarrhea…”* (VET14). Overall, most of the veterinarians mentioned pain as a crucial factor when assessing QoL for dogs in general and for dogs with CE: *“… for the dog, I think, if it is in pain”* (VET14). Three veterinarians used the canine chronic enteropathy clinical activity index (CCECAI) (26) or the canine inflammatory bowel disease activity index (CIBDAI) (27): *“… (the index) gives an objective assessment… and at the same time, it reflects how the owner sees things”* (VET15). These composite scores include variables such as activity level, attitude, appetite, weight loss, presence and frequency of vomiting, diarrhea, etc. They were created to assess disease severity and prognosis but have not been constructed nor validated to assess QoL (26, 27).

##### 3.2.2.3 Emotional state of the dog

The emotional state of the dogs was assessed based on an owner history, as well as on the veterinarian’s interpretation of the history, *“The happiness of the dog is the most important thing to me.”* (VET1). The emotions that were most commonly mentioned by the veterinarians included joy and happiness, but also shame: *“…I think many of them will feel ashamed”* (VET1). They also mentioned a loss of dignity: *“That is the thing with dignity, they (the dogs) know when they did something wrong”* (VET7). Some veterinarians used just one of these approaches, while others used a combination: *“…then I will talk a lot with the owners and then perform the physical examination and the laboratory analyses as well”* (VET17). However, regardless of the approach, many veterinarians referred to the importance of good dialogue with the dog owner as the foundation for the QoL assessment, e.g., *“Initially, I talk to the owner…”* (VET12). This relates to veterinarians relying heavily on a history from the dog owner combined with their own clinical assessment.

### 3.3 Theme 2: owner reflections on the multifaceted impact of having a dog with CE

The majority of owners had many concerns and experienced burdens in relation to having a dog with chronic GI disease, which impacted their own QoL. These could be divided into: (i) daily logistical challenges, (ii) implementing therapeutic regimens such as diet restriction and administering multiple daily medications, and (iii) the strain of nursing responsibilities on the owner-dog relationship.

#### 3.3.1 Daily logistical challenges of having a dog with CE

The effect on everyday logistics ranged from having no impact on the owners’ lives and routines to considerable time and effort spent on the dog’s health, to controlling the owner’s life completely. One owner found a practical solution that did not have a considerable impact on their daily routines: *“… a dog that had to eat four times a day… I managed this with an automatic pet feeder”* (DO3). However, several dog owners described how having a dog with CE had affected their holiday plans and daily work life: *“…there are places that you cannot take her. Then you need someone to pet-sit her, again.”* (DO15). Several owners described having to take an occasional day off work to take care of their dog and the disappointment on returning to work and still having a sick dog: *“I would come home from work and he had been sick. I would ask myself, why did I go to work?!”* (DO11). Others described how they found it difficult or were unable to maintain normal working hours because they worried about their dog’s wellbeing, e.g., *“I could not go anywhere, because he felt poorly”* (DO5)*, “… when she was very sick, I could not go to work. It was emotionally hard, I felt powerless and irritated”* (DO3). Many owners described how they had to plan ahead a lot more in terms of social engagements or simply going shopping, as their dog could not be left alone. Finally, some owners had to cancel day trips and holidays: *“… holidays need to be (carefully) planned because someone has to stay at home to take care of her (the dog)”* (DO15). One owner was unable to visit her daughter in France due to the dog’s illness. In addition, two owners reported that they bought surveillance cameras to monitor their dogs while at work.

#### 3.3.2 Implementing dietary restrictions and medications

Dietary restrictions were often described as a part of the everyday logistical challenges. Several owners described in detail how they learned to adhere to a complex feeding plan with several specified meals a day: *“… she is fed twice daily, 45 grams in the morning, 45 grams in the evening and 20 grams of treats”* (DO15). However, they also described the demands involved in testing a new diet, potentially several times, the expense of prescription diets and buying different types of food for diet trials, as well as the demands related to adapting to a restrictive behavior with regard to other commercial foods. Furthermore, the constant focus on feeding their dog a specific diet and ensuring that the dog did not ingest any other food or treats by accident added extra pressure and guilt to the owner’s life, e.g., *“now we are totally hysterical about what he gets and have been very strict about his diet”* (DO16). One area where a number of owners felt frustrated was their inability to feed or find suitable treats. Treats often serve as positive reinforcement in a training situation, e.g., *“I’m standing there, cutting these treats into four parts so they can at least last for a training session of 10 min”* (DO6). However, treats were also involved in owner-dog bonding situations, where feeding in general played a vital part: *“… (the dog) wants a treat, and then we are pals, but then again (the dog) will have diarrhea for 4 days”* (DO14). Several owners described how they had searched the market for treats that their dog could eat despite having CE, but without much luck: *“… we must find other alternatives, but… there is not a bone in this world that she can tolerate”* (DO13).

Conversely, owners appeared to deal with the need for medical therapy well: *“… medicine is not especially difficult to administer”* (DO18). They often mastered administering multiple types of medication and other kinds of care with little effort: *“I have also figured out that leaving a warm compress on his belly will calm him down”* (DO5).

#### 3.3.3 Emotional consequences of having a dog with CE

Owners reported that at the time of the interview they generally had much more focus on even small changes in the dog’s condition and needs, including for example monitoring fecal consistency. Several owners explained that they monitored their dogs around the clock, that the dog’s well-being was always on their mind, and that it was a stress factor, e.g., *“… it is a huge stress factor, everything must be as stress-free as possible. One is constantly awaiting some kind of reaction”* (DO13). Owners reported how having a dog with clinical signs of CE had been emotionally hard to deal with when the dog experienced active clinical signs, but also afterwards when monitoring the dog for a potential relapse, *“I’m so worried that I’m not doing a good enough job. I really want a dog that’s able to play, but he is not doing that very much”* (DO2). The owners described their emotional state with adjectives such as stress, worry, being unhappy, and feeling powerless, e.g., *“Every time he made even the smallest sound, we were very stressed. Particularly me, I have been very affected”* (DO10).

### 3.4 Theme 3: owner-veterinarian communication and relationship

#### 3.4.1 Owner perspective

Owners described (i) their general perspectives on communication with their veterinarian and (ii) that the complicated nature of CE leads to intensified veterinary contact and communication.

##### 3.4.1.1 Generally good communication, with some caveats

The majority of owners (15/20) reported that they had confidence in and good communication with their veterinarians: *“… they have been very good at answering questions, it’s a good collaboration”* (DO10). Six owners responded that they had encountered some veterinarians who listened to them, *“… overall, I think that we have had really good communication”* (DO1), as well as some who did not. Examples of the latter could be if the owners felt that the veterinarians did not take their complaints seriously or did not know enough about the disease. One owner mentioned that the veterinarian did not appear to be very empathetic of the influence that the dog’s disease had on their life: *“I did not experience the same degree of understanding of what we (as dog owners) actually go through”* (DO13). One owner experienced poor communication with a couple of veterinarians, and actively did not want to follow their advice with regard to treatment. Owners also reported that, at larger practices, they might see several different veterinarians, which sometimes made communication more difficult due to the different responses from the different veterinarians, e.g., *“… at this point another veterinarian started interfering a lot… so since then, we have asked to be seen by the same veterinarian at each visit”* (DO11). Owners reported that seeing different veterinarians out of hours (e.g., practice collaborations covering night shifts) was challenging and required more elaborate communication as these veterinarians did not necessarily have access to the dog’s medical records or information about the diagnostic work-up or final diagnosis: *“I think everything happened too fast and that there were too many (veterinarians) involved”* (DO18). This made alignment in diagnostics and treatment challenging, and resulted in frustrated clients when the new veterinarian said something that contradicted the first, or when the new veterinarian simply did not have access to previous test results and notes.

##### 3.4.1.2 Density of CE information is challenging for dog owners

Owners generally reported that the disease and necessary diagnostic work-up was explained in detail by their own veterinarians. However, this information was considered relatively complex and difficult to comprehend, and owners would therefore often seek further information themselves and had additional follow-up queries: *“I think that there was too much scientific language, and I had to go home and Google it”* (DO13). Several owners (9/20) reported that the veterinarians urged them to get in touch if they had further questions, and the owners frequently made use of this service. This typically included telephone calls, contact via social media, or direct emails to the veterinarian. Most owners (18/20) reported that all their queries were answered: *“I actually think that veterinarians are easier to communicate with compared to most human physicians”* (DO16).

#### 3.4.2 Veterinarian perspective

Several veterinarians described how they found communication with owners of CE patients laborious for a number of reasons, including: (i) CE patients require an extensive work-up to reach a diagnosis, (ii) the specific therapy was often based on trial-and-error, which could frustrate the client, and there would often be a number of follow-up visits, and (iii) many veterinarians described multiple follow-up queries that resulted in non-billable hours spent on email correspondence and telephone calls.

##### 3.4.2.1 Communication regarding CE work-up

Several veterinarian participants referred to the complexity of CE in dogs as a reason why more time and effort was spent communicating with owners for this patient group: *“… this is crazy complicated stuff… that makes it really difficult to communicate”* (VET1). They found it difficult to communicate the need for diagnostics requiring a stepwise approach with multiple tests over a longer period of time that are both time consuming and costly: *“… then I spend a lot of time explaining why there are so many necessary tests”* (VET14). This complexity could create challenges in the veterinarian-owner relationship: *“… it can be a long and hard process and of course the owners can become frustrated”* (VET16). Most veterinary participants found themselves having to spend much more time than expected explaining the diagnostic and therapeutic approaches, e.g., *“(The owners) think that diarrhea is a disease, not a clinical sign”* (VET1).

##### 3.4.2.2 Dietary management based on trial and error

Many veterinarians specifically mentioned challenges regarding diet and treats in relation to treatment, *“Diet is an area that preoccupies the owners immensely. This is an area where I can experience issues with compliance”* (VET17). Veterinarians often described poor owner compliance when prescribing a specific hypoallergenic diet, where it was of utmost importance that the client adhered to giving the dog only this diet for a specified number of weeks: *“If I put the dog on a hydrolyzed diet, I know that there may be compliance issues that I need to deal with before the owners leave”* (VET19).

##### 3.4.2.3 Responding to owner queries

The veterinarians observed that due to the extensive work-up and challenging treatment regimens, owners of dogs with CE would contact them more often compared to owners of other types of veterinary patients. Therefore, some veterinarians would often use alternative communication approaches in addition to the traditional verbal communication during the consultation. This mostly involved phone calls or emails, e.g., *“I tell them to call in a week, or send a picture, and then we can communicate via email”* (VET18). A number of veterinarians provided written explanatory material, diagnostic approach tables, or medication schedules. However, several veterinarians described how they would have liked to provide the dog owners with written material as an adjunct to verbal information, but due to the complexity and difference in disease presentation between individual dogs as well as a lack of time, this was not feasible: *“I have a lot of plans about creating written material… it is just so difficult, because no two patients are alike”* (VET1). Therefore, it appears that most of the communication was verbal. Much of the additional communication was considered “non-billable hours” and the veterinarians would rarely find the necessary time to provide this additional communication within normal business hours.

## 4 Discussion

### 4.1 Theme 1: the understanding and assessment of quality of life for dogs with chronic enteropathy

When assessing QoL, owners mostly focused on behaviors that showed the presence of disease, while veterinarians considered QoL for dogs with CE on one or more of the following three sub-themes: presence or absence of normal behavior, the degree of disease severity, and the emotional state of the dog. Veterinarians assessed this by interpreting the history given by the owner and evaluating the dog during the consultation.

Veterinarians listed pain as the most important parameter for determining QoL, while this was the second or third most important parameter for dog owners, who instead found vomiting to be the most important parameter. Vomiting is an easily recognizable clinical sign for lay persons, while behavior associated with (abdominal) pain might be more difficult to assess in dogs. Assessment of pain is an integral part of a clinical consultation and one of the parameters that must be considered (according to national animal protection legislation) by veterinarians when making decisions about continuing treatment. It is therefore not surprising that the veterinarians selected this parameter (28).

When assessing QoL, owners generally used more human-derived adjectives and emotional terms. Veterinarians seemed to struggle to define QoL for dogs with CE and would often use clinical signs as indicators and human-derived adjectives only to a minor degree. There is no generally accepted consensus for the definition of QoL in dogs (29, 30). However, QoL assessment systems do exist, and while these may be beneficial for a general assessment of dogs’ health (31–33), they may lack the more specific evaluation of clinical signs and therapeutic intervention related to a particular disease. Marchetti et al. attempted to tailor a QoL assessment specifically for CE using owner questionnaires (18). This QoL assessment includes five themes: “general,” “health,” “activity,” “interaction,” and “stimulation.” Its format may be too extensive to allow for evaluation in a standard clinical context, but it could potentially be of use in a reduced format. However, no veterinarians in the current study mentioned using this system or other published QoL questionnaires or QoL scoring systems for dogs in general (18). Instead, a couple of veterinarians used CCECAI or CIBDAI as indicators of QoL in dogs with CE. These semi-objective indices were originally developed as research tools to assess disease activity, remission, and progression in dogs with CE (26, 27), and have, to a certain extent, been used in clinical practice. They include parameters such as activity level, appetite, frequency of vomiting, fecal consistency, and defecation frequency (26, 27). The indices were not validated to assess QoL, but rather to assess risk factors for a negative outcome (26). There appears to be a need for simple and validated assessment tools to assist veterinarians in clinical practice when assessing pain and other aspects of QoL for dogs with CE.

In general, owners and veterinarians were relatively aligned when describing how they viewed QoL for dogs with CE, and the majority of veterinarians relied heavily on the owners’ input and observations.

### 4.2 Theme 2: the multifaceted impact of having a dog with CE

Owners of dogs with CE were faced with several challenges specifically related to daily logistical challenges, dietary trials, treatment management, as well as the emotions related to having a sick dog.

Logistical challenges of having a dog with a chronic GI disease often lead to lifestyle changes for the owners, including changing or reducing working hours and cancelling holidays or other social engagements. Similar findings were identified for owners of dogs with idiopathic epilepsy, where owners experienced social isolation due to cancelling or avoiding social events to take care of their dogs (34).

Studies of dogs with protein losing enteropathy, a subtype of CE often associated with a worse outcome, showed that dogs were more likely to come out of remission if their owners could not adhere to the dietary recommendations (35). While owners in the present study reported that the dogs with CE would often eat the prescribed diet and the owners themselves would quickly become adept at administering medications, restrictions in diet and treats were still a subject of great concern. Although owners rationalized that their dogs were “just dogs,” they would still feel sorry for them because they had to eat the same food all the time. In alignment with these findings, it has previously been shown that dog owners may appear resistant to changes in their dogs’ diet, depending on their pet’s food preferences and if it is a multi-pet household (36). Non-compliance is known to be a great challenge in obesity programs for example, where approximately half of owners will not adhere to a feeding protocol for their pets (37). The owners’ behavior and degree of compliance may change depending on their veterinarian’s behavior. However, when veterinarians initially take time to gather relevant diet- and patient-related information and educate their clients about the benefits of the new diet, owners are more prone to follow the advice (36, 38). Interestingly, qualitative studies examining the opinions and experiences of veterinarians regarding the recommendations and use of prescription diets and treats could not be identified. In this study, treats and chews were a particular challenge, as owners needed these for training situations, for positive reinforcements in general, and to maintain owner-dog bonding. This finding is supported by previous studies where it was evident that treats represented a conspicuous part of the dog’s diet (39–41). While some owners searched the market for non-allergenic treats, others simply expressed their frustration. Veterinarians could also confirm this frustration, as they too would often perceive owners to be frustrated with having to stick to a specific diet and the restricted use of treats. Giving the client options for hypo-allergenic treats and alternatives at the initial consultation could alleviate some of this frustration and potentially improve compliance.

Studies have previously shown that having a dog with a severe chronic disease has a profound impact on the owner and that this burden is exacerbated for both dog and cat owners the longer the illness is present (21). In addition, studies examining owners of dogs and cats with various chronic diseases have shown that the level of disease burden in their pet correlated with the owners’ level of stress and depression and a reduction in their QoL (21). Furthermore, a previous study on idiopathic epilepsy showed that a reduction in a dog’s QoL was strongly related to a reduction in their primary caregiver’s QoL (42). Considering this added effect of owner burden and potential for psychosocial challenges for the owner, effective veterinarian-owner communication here seems vital (43).

Overall, it appeared that having a dog with CE had a considerable impact on several aspects of the owners’ life, which in turn affected the owner’s QoL.

### 4.3 Theme 3: owner-veterinarian communication and relationship

Owners interviewed about communication with their veterinarian reported that it was generally good and they had multiple points of contact with their veterinarian due to the complicated nature of CE. In contrast, veterinarians reported that communication was often laborious due to the complicated nature of the condition, the extensive diagnostic work-up, and potential owner frustrations related to treatment trial and error, resulting in a high number of non-billable hours. Owners were generally content with their veterinarians, but they did find that dealing with the emotional stress of having a sick dog, the diagnostic work-up, and seeing different veterinarians at frequent check-ups and feeding trials to be quite an ordeal. Likely also related to the complexity of the disease, owners would generally contact their veterinarians with questions regarding their dog’s immediate health, questions relating to understanding the disease, or practical queries regarding treatment. Veterinarians made themselves available via different contact means, and it was clear that there would often be frequent communication between owners and veterinarians during the diagnostic work-up and until the dog was in remission. Veterinarians confirmed this finding, revealing that this type of disease would lead to multiple contact points with the owners. Previous studies examining successful communication between veterinarians and owners identified several specific focus areas. These included client education, which should be presented in different formats and offer the owners choices, using two-way communication. Barriers to good communication were often related to not listening to the owners, misinformation and not providing all available options, as well as financial constraints and time limitations (23, 44). In our study, the majority of veterinarians did not provide a written discharge instruction and several veterinarians considered using this to optimize communication. Several veterinary participants rejected this due to a lack of sufficient time and the need for tailored information for the individual dog between consultations. Although it was not specifically assessed in our study, providing detailed written information to clients could potentially minimize the number of interactions and non-billable hours, and this should be explored in future studies.

In general, communication between owners and veterinarians seemed to be good. However, we found areas where veterinarians could improve, notably by giving written instructions, thereby potentially improving compliance.

## 5 Limitations

One limitation of the methods employed in this study is that owners and veterinarians were not paired. The authors of this study could have interviewed owners who used one of the veterinarians interviewed in the study as their local practice, but such owner-veterinarian pairings were not pursued. In addition, the interviewers were relatively inexperienced in interview techniques, and it is likely that questions were asked in a more nuanced manner as the interviewers acquired more skills during the study. Furthermore, veterinarians were recruited through networking rather than volunteering. Although the authors attempted to have a balanced selection of veterinarians from different geographical regions and postgraduate experience, the recruitment method could give rise to bias, thus missing important perspectives. All interviews were conducted and transcribed in Danish, which was the native language. The selected quotes used in this manuscript were translated into English by JGHL and LNN (Supplementary file S3), but it is possible that some more subtle meanings and cultural understandings were lost in translation.

## 6 Conclusion

In conclusion, CE in dogs affects the QoL of both the dogs and their owners. While the assessment of QoL by owners and veterinarians was aligned, veterinarians seemed to lack easily applicable tools to assess QoL in this patient group.

Having a dog with CE negatively affected the owner’s QoL and posed daily logistical challenges. This specifically included dietary trials and treat restriction, which many owners found difficult to manage. In addition, managing the dogs’ treatment, as well as all the emotions related to having a sick dog negatively impacted the owners’ QoL.

Owners generally felt that communication with their veterinarian was good, while veterinarians found the communication laborious and time-consuming. Providing owners with written material about the condition as well as medical and feeding regimen instructions may help the owner, improve compliance, and reduce the non-billable hours the veterinarians spend communicating with owners about CE in their dogs.

## Data Availability

The raw data supporting the conclusion of this article will not be publicly available to protect participant confidentiality and privacy. Requests to access these datasets should be directed to the corresponding author.
