# Supplementary material for: Chronic enteropathy in dogs affects the quality of life in both dogs and their owners—are veterinarians proficient in handling the caregiver burden?
Source: Front Vet Sci. 2025 Jan 7;11:1488917. doi: 10.3389/fvets.2024.1488917 (PMC11747720; doi:10.3389/fvets.2024.1488917)
Supplement: Supplementary file 1 [file Data_Sheet_1.docx]

**Supplementary file 1**

**Interview guide - Dog owners:**

**Open questions:**

- What course of disease have you and your dog experienced up until now?
- How has your dog’s disease affected your everyday life?
- How do you assess your dog's quality of life (how do you determine if your dog is well/having a good dog life)?

**Follow-up/ closed questions:**

- What do you (mainly) base your assessment of the dog's quality of life on?
- How do you think your dog is doing on a daily basis?
- Have you experienced, that your dog exhibits any of the following symptoms (see table) in daily life, and which do you feel affect the dog's quality of life the most?:

| *Diarrhea* |  | *Vomiting* |  |
| --- | --- | --- | --- |
| *Anorexia*  *(do not want to eat)* |  | *Pain* |  |
| *Restlessness* |  | *Weight loss* |  |
| *Tenesmus*  *(Tries to defecate, but to no avail)* |  | *Changing behavior*  *(E.g. increased socializing, self-isolation, etc.)* |  |

- How big of a problem is your dog's illness for you?
- Has it affected vacation plans/social life/personal life?
- Does having a dog with a chronic gastrointestinal disorder cause you daily worries/stress?
- What treatments have been initiated, and how often does the dog have follow-up examinations with the veterinarian?
- How is the execution of the treatment(s) going?
- Does the treatment affect your daily life?
- How have you experienced the visits and communication with the veterinarians who have been part of your process?

Communication with the veterinarian:

- Do you feel that the veterinarian listened to your problems/thoughts?
- Do you feel that you were involved in the decision about the treatment plan?
- Were all of your questions answered during/after the consultation?
- Do you feel you received all the information you needed? Did you understand all the information?
- Did you need to seek information elsewhere afterward?
- Is there anything you wish the veterinarian had done/does differently? (information, treatment, examination)

**Interview guide - Veterinarians**

**Open questions:**

- What do you focus on when assessing the quality of life in a dog in general?
- What parameters do you use to assess the quality of life in a dog with chronic enteropathy?
- Do you experience any problems when communicating with clients about dogs with chronic enteropathies, and if so, what are they?
- Are there any special considerations you make when communicating with the owner of this type of dog – do they have special needs, are there specific focus areas?

**Follow-up/closed questions:**

Quality of life:

- Which clinical symptoms do you find most significant for the dogs' quality of life?
- Do you find that there can be differences between your perception and the clients' perception of the dog's quality of life?

Communication with owners:

- Do you experience problems with client compliance?
- Do you find it problematic that clients do not understand the information you give them?
- In your opinion, what is the best way to convey information to the owner?
- Do you find that dog owners have sought information on the internet (or elsewhere) before/during the consultation with you?
