# Supplementary material for: Chronic enteropathy in dogs affects the quality of life in both dogs and their owners—are veterinarians proficient in handling the caregiver burden?
Source: Front Vet Sci. 2025 Jan 7;11:1488917. doi: 10.3389/fvets.2024.1488917 (PMC11747720; doi:10.3389/fvets.2024.1488917)
Supplement: Supplementary file 2 [file Data_Sheet_2.docx]

Supplementary file 2

| **Code book – Veterinarian interviews** | | |
| --- | --- | --- |
| **Codes:** | **Sub-codes:** | **Examples:** |
| QoL | The quality of life in dogs in general. | Different methods for assessment of QoL such a clinical signs i.e. appetite, results of specific tests such as blood test or clinical scores, or just general considerations on QoL in dogs. |
|  | The QoL in dogs with GI disease. | (same as above but only statements relating specifically to dogs with GI disease) |
|  | How does the veterinarians perceive the QoL of the owners? | Influence on owner-dog relationship, influence on everyday life of the owner. |
|  |  |  |
| Communication | Is there an alignment of expectations between veterinarians and the owner, and diagnostic work up performed by the vet? | If the vet considered chronic GI disease a “difficult/challenging” subject, thoughts about preparing the client for a long diagnostic workup |
|  | How does the veterinarian do follow up including amount of time used? | This includes physical re-checks, and oral or written communication, as well as comments on time or energy used on follow up. |
|  | Do vets experience that owners are seeking knowledge from elsewhere and how do they deal with it? | Owner seek information on the internet/ social media/ breeders/ friends and family |
|  | Do vets find it difficult to get owners to use specific diets as therapy, are owners compliant? and do owners understand why they have to give it/why are they not compliant? | “it is difficult to the client to do a strict diet trial” |
|  |  |  |
| Owner resources | Does the veterinarian treat people based on their perception of owner situation such as socio-economic, level of education, or personal resources? | The vet does not offer expensive work-up because they know the client has low income |
|  | Do veterinarians have an opinion about the costs for the owner with regards to dogs with gastrointestinal disease? | Expensive diagnostics, treatments, complaints (ie owners complain that the tx is expensive) |

| **Code book – Owner interviews** | | |
| --- | --- | --- |
| **Codes:** | **Sub-codes:** | **Examples:** |
| QoL | From the owners perspective, how is their dog's quality of life. | Owners telling of clinical signs, behavioral changes etc. E.g. if the dog is not eating or sadness |
|  | Dog-owner interaction and relationship - both how they physically interact and how the owner emotionally connect with the dog. | The dog will be "coming for cuddles", being responsive to the owner talking to it etc. |
|  | Considerations on owner QoL | What is the impact on the owner´s life? does the owner worry? Have to cancel vacations etc. |
|  |  |  |
| Communication | Veterinarian–owner interaction and relationship, interactions, and how well is their relationship? | How does the veterinarians and owners interact? phone call, talk, write, meet  Relationship with trust/ distrust/respect etc. |
|  | Sources of information used by the owner | Other veterinarians, Google, specific web-pages |
|  |  |  |
| Owner resources | Comments related to costs (this includes only costs in relation to diagnostics, rechecks, coping with chronicity. |  |
|  | Comments related to diet | Thoughts on giving a specific diet long term to their dog. |

QoL – Quality of Life
