# Supplementary material for: Chronic enteropathy in dogs affects the quality of life in both dogs and their owners—are veterinarians proficient in handling the caregiver burden?
Source: Front Vet Sci. 2025 Jan 7;11:1488917. doi: 10.3389/fvets.2024.1488917 (PMC11747720; doi:10.3389/fvets.2024.1488917)
Supplement: Supplementary file 3 [file Data_Sheet_3.docx]

Supplementary file 3 – Original and translated quotes

| **Original Danish quote** | **Translated quote** | **Informant** |
| --- | --- | --- |
| **Theme 1** |  |  |
| ***Owners:*** |  |  |
| *”Hun er glad, hun træner, så på den måde er hendes livskvalitet rigtig god.”* | *“She is happy, she is training, so in that sense her QoL is really good”* | *DO11* |
| *”…han kan jo sagtens lege fangeleg…”* | *“… he can easily play a game of tag”* | *DO14* |
| *“…han har virkelig, altså trukket sig væk fra os og passede sig selv og ikke rigtig, altså været meget træt og bare havde lyst til at være i fred og ro.”* | *“… he has really withdrawn himself from us, he’s minding his own business, he’s been tired and has only wanted peace and quiet.”* | *DO8* |
| *“Kan jeg få hende til at lege…fordi det kunne jeg ikke I starten. Hun ville ikke lege, hun ville ingenting.”* | *“Can I make her play… because I couldn’t at the beginning. She didn’t want to play; she didn’t want to do anything.”* | *DO11* |
| *”…den der med at hun hele tiden er under opsyn, man har ikke frihed”* | *“… she is constantly under supervision, that way, you don’t have any freedom.”* | *DO12* |
| ***Veterinarians:*** |  |  |
| *“Jeg går meget op I, om hunden har den adfærd, som den altid har haft, er det de samme ting, som gør den glad.”* | *“It is important to me that the dog has the same behavior that it has always had.”* | *VET1* |
| *“…frekvens, grad af opkast og diarré…”* | *“…frequency and severity of vomiting and diarrhea…”* | *VET14* |
| *“…for hunden tænker jeg, (om) den har smerter”* | *“… for the dog, I think, if it is in pain”* | *VET14* |
| *“… (indekset) giver en objektiv vurdering…. og samtidig er det jo hvordan ejeren ser det”* | *“… (the index) gives an objective assessment… and at the same time, it reflects how the owner sees things”* | *VET15* |
| *“Hundens glæde er det vigtigste for mig.”* | *“The happiness of the dog is the most important thing to me.”* | *VET1* |
| *“…jeg tror…mange af dem…vil føle skam”* | *“…I think many of them will feel ashamed”* | *VET1* |
| *“det er jo det med værdighed,…de ved godt at det var forkert, det de har gjort”* | *“That is the thing with dignity, they (the dogs) know when they did something wrong”* | *VET7* |
| *“så snakker jeg meget med ejeren….og så klinisk undersøgelse…og paraklinikken også”* | *“…then I will talk a lot with the owners and then perform the physical examination and the laboratory analyses as well”* | *VET17* |
| *“I første omgang samtale med ejeren…”* | *“Initially, I talk to the owner…”* | *VET12* |
| **Theme 2** |  |  |
| *“…en hund der skulle spise fire gange om dagen…det fik jeg løst med en foderdispenser”* | *“… a dog that had to eat four times a day... I managed this with an automatic pet feeder”* | *DO3* |
| *”…der er …steder, man ikke kan have hende med…så skal man igen have en til at passe.”* | *“…there are places that you cannot take her. Then you need someone to pet-sit her, again.”* | *DO15* |
| *”man kom hjem fra arbejde….så havde den kastet op..hvorfor tog jeg på arbejde?!”* | *“I would come home from work and he had been sick. I would ask myself, why did I go to work?!”* | *DO11* |
| *“jeg kunne slet ikke tage nogle steder, fordi han havde det dårligt”* | *“I couldn’t go anywhere, because he felt poorly”* | *DO5* |
| *“…da hun var meget syg, der kunne jeg ikke arbejde, det var følelsesladet, …afmagt og irriterende* | *“… when she was very sick, I couldn’t go to work. It was emotionally hard, I felt powerless* *and irritated”* | *DO3* |
| *”…ferier skal planlægges, fordi der skal være en hjemme og passe hende”* | *“… holidays need to be (carefully) planned because someone has to stay at home to take care of her (the dog)”* | *DO15* |
| *”hun får foder to gange om dagen, 45 gram om morgenen, 45 gram om aftenen og 20 gram godbidder vejet“* | *“… she is fed twice daily, 45 grams in the morning, 45 grams in the evening and 20* *grams of treats”* | *DO15* |
| *”nu er vi helt hysteriske med hvad han får, altså…været meget strikse med vores diæt”* | *“now we are totally hysterical about what he gets and have been very strict about his diet”* | *DO16* |
| *”jeg står og skærer de der madknapper ud I 4 dele for at det overhovedet kan vare til en træning på 10 minutter”* | *“I’m standing there, cutting these treats into four parts so they can at least last for a training session of 10 minutes”* | *DO6* |
| *”den skal have en godbid, så er vi gode venner, men så har vi.. diarré i 4 dage.”* | *“… (the dog) wants a treat, and then we are pals, but then again (the dog) will have diarrhea for 4 days”* | *DO14* |
| *”vi må finde andre alternativer, men et kødben…findes ikke I den her verden, som hun kan tåle”* | *“… we must find other alternatives, but… there is not a bone in this world that she can tolerate”* | *DO13* |
| *“medicine er ikke noget..specielt at give“* | *“… medicine is not especially difficult to administer”* | *DO18* |
| *”Har også fundet ud af, at jeg lægger varme omslag på hans mave..så falder han egentlig godt til ro, når han får de ting“* | *“I have also figured out that leaving a warm compress on his belly will calm him down”* | *DO5* |
| *”…det er en kæmpe stressfaktor…alting skal være så stressfrit som muligt og man går næsten og venter på en reaktion”* | *“… it is a huge stress factor, everything must be as stress-free as possible. One is constantly awaiting some kind of reaction”* | *DO13* |
| *”jeg er simpelthen så bange for at man ikke gør det godt… jeg vil så gerne have en hund der kunne lege, for det gør han ikke meget”* | *“I’m so worried that I’m not doing a good enough job. I really want a dog that’s able to play, but he is not doing that very much”* | *DO2* |
| *“Vi selv har været meget stressede, hver gang han har sagt et lille piv.. jeg har særligt været meget påvirket”* | *“Every time he made even the smallest sound, we were very stressed. Particularly me, I have been very affected”* | *DO10* |
| **Theme 3** |  |  |
| ***Owner perspective:*** |  |  |
| *“de har været rigtig gode..til ..at svare på spørgsmål, godt samarbejde“* | *“… they have been very good at answering questions, it’s a good collaboration”* | *DO10* |
| *“som udgangspunkt synes (jeg)..vi har haft en rigtig fin dialog”* | *“… overall, I think that we have had really good communication”* | *DO1* |
| *“der var det sådan at jeg ikke oplevede den helt samme form for forståelse for, hvad vi faktisk havde været igennem“* | *“I did not experience the same degree of understanding of what we (as dog owners) actually go through”* | *DO13* |
| *“der var der så en anden dyrlæge over, og som så også begyndte at blande sig en hel masse… så siden da, der har vi bare kørt med at vi ligesom har bedt specifikt om at komme til den samme dyrlæge.”* | *”… at this point another veterinarian started interfering a lot… so since then, we have asked to be seen by the same veterinarian at each visit”* | *DO11* |
| *“jeg tror det går for stærkt, der er for mange (dyrlæger) inde over”* | *“I think everything happened too fast and that there were too many (veterinarians) involved”* | *DO18* |
| “jeg syntes at der har været meget I fagtermer og så har jeg selv måttet gå hjem og google noget” | *“I think that there was too much scientific language, and I had to go home and Google it”* | *DO13* |
| “jeg synes faktisk at dyrlæger er lettere… at kommunikere med end de fleste læger” | *“I actually think that veterinarians are easier to communication with compared to most human physicians”* | *DO16* |
| ***Veterinarian perspective:*** |  |  |
| *“…det er sindssygt kompliceret stof..det gør det virkelig svært at videreformidle”* | *”… this is crazy complicated stuff… that makes it really difficult to communicate”* | *VET1* |
| *“…så bruger jeg meget tid på at kommunikere. hvorfor så mange prøver er nødvendige”* | *“… then I spend a lot of time explaining why there are so many necessary tests”* | *VET14* |
| *“**…det kan være hårde lange forløb...selvfølelig kan…ejere være meget frustrerede”* | *“… it can be a long and hard process and of course the owners can become frustrated”* | *VET16* |
| *“De (ejere) synes at diarré er en sygdom, …ikke..et symptom.”* | *“(The owners) think that diarrhea is a disease, not a clinical sign”* | *VET1* |
| *“fodring…helt personligt fylder det meget for dem (ejerne), der kan jeg godt opleve problemer compliancemæssigt”* | *“Diet is an area that preoccupies the owners immensely. This is an area where I can experience issues with compliance”* | *VET17* |
| *“Hvis man sætter den på en hydrolyseret diæt…den compliance skal jeg tage hånd om, når de går herfra”* | *“If I put the dog on a hydrolyzed diet, I know that there may be compliance issues that I need to deal with before the owners leave”* | *VET19* |
| *“jeg siger til dem… ring lige om en uge … eller send et billede….så kommunikerer vi via mail”* | *“I tell them to call in a week, or send a picture, and then we can communicate via email”* | *VET18* |
| *“jeg har mange planer om at lave det skriftligt…det er bare så svært fordi der…ikke er to patienter som er ens”* | *“I have a lot of plans about creating written material… it is just so difficult, because no two patients are alike”* | *VET1* |

DO – dog owner; VET - veterinarian
